# Species distribution and antimicrobial susceptibility profiles of nontuberculous mycobacteria, with an exploratory 12-month completer outcome analysis: a single-center retrospective study from Shandong Province, China

**DOI:** 10.3389/fmicb.2026.1917292

**Published:** 2026-10-20

**Authors:** Jie Zhang, Xiao Ru, Yuming Shao, Li Xu, Hengjian Fan

**Affiliations:** 1 Department of Critical Care Medicine, Shandong Provincial Maternal and Child Health Care Hospital Affiliated to Qingdao University, Jinan, Shandong, China; 2 Department of Tuberculosis Medicine, Public Health Clinical Center Affiliated to Shandong University, Jinan, Shandong, China; 3 Department of Breast and Thyroid Surgery, Shandong Provincial Maternal and Child Health Care Hospital Affiliated to Qingdao University, Jinan, Shandong, China; 4 Department of Respiratory Endoscopy, Public Health Clinical Center Affiliated to Shandong University, Jinan, Shandong, China; 5 Department of Respiratory and Critical Care Medicine, Public Health Clinical Center Affiliated to Shandong University, Jinan, Shandong, China

**Keywords:** antimicrobial susceptibility testing, China, clinical breakpoints, minimum inhibitory concentration, *Mycobacterium avium* complex, nontuberculous mycobacteria, real-world evidence

## Abstract

The incidence of nontuberculous mycobacterial pulmonary disease (NTM-PD) is rising worldwide, yet clinical breakpoints validated against treatment outcome exist for very few antimycobacterial agents. We characterized species distribution and broth-microdilution minimum inhibitory concentration (MIC) profiles in 606 consecutive isolates, one per patient, collected between February 2021 and October 2024 at a provincial referral hospital in Shandong Province, China. Outcomes were examined in the 127 patients (21.0% of all 606) who received at least 12 months of therapy. Testing followed CLSI M24 (3rd edition). The panel reports MIC values only and the laboratory assigns categories, having revised its breakpoints twice as successive CLSI standards were adopted. To apply one criterion throughout, the four agents with CLSI clinical breakpoints for the *Mycobacterium avium* complex (MAC), namely clarithromycin, amikacin, linezolid and moxifloxacin, were reclassified from individual MIC values. MAC accounted for 73.1% of isolates, the *Mycobacterium abscessus* complex (MABC) for 12.4% and *Mycobacterium kansasii* for 8.1%. Against MAC, 98.9% of isolates were susceptible to clarithromycin, 99.8% to amikacin, 99.3% to linezolid and 96.2% to moxifloxacin. Azithromycin was assigned to a higher category than clarithromycin in 97 isolates, and 93 of these remained in the lowest clarithromycin category; this difference reflects the thresholds applied to the two macrolides rather than independent resistance. Ethambutol MICs were concentrated at the upper end of the tested range (44.6% at the highest concentration), which describes the MIC distribution rather than resistance, because no clinical breakpoint exists for ethambutol against MAC. Yearly distributions showed no monotonic trend (*P* = 0.063). Cure was achieved in 58.8% of MAC-PD, 54.5% of MABC-PD and 95.0% of *Mycobacterium kansasii*-PD patients; these estimates describe the completer cohort only. Cavitary disease was associated with substantially lower odds of cure (adjusted odds ratio 0.15, 95% CI 0.04–0.54, *P* = 0.004), whereas regimen composition was not. Susceptibility testing is actionable for the macrolides and amikacin, with clarithromycin the appropriate macrolide class representative; for other agents the categories depend on locally defined criteria that changed during the study. NTM surveillance should therefore be built on MIC values, and should record the interpretive criteria applied.

## 1 Introduction

Nontuberculous mycobacteria (NTM) comprise more than 200 recognized species and subspecies, approximately 60 of which cause human disease (Griffith et al., 2007). Pulmonary involvement predominates, although lymphadenitis, skin and soft-tissue infection and disseminated disease in immunocompromised hosts are increasingly recognized (Xu et al., 2024). Reported incidence has risen across high- and middle-income countries over the past decade (Winthrop et al., 2020; Ko et al., 2018), while advances in molecular and proteomic identification have improved diagnostic resolution and revealed marked geographical structure in species ecology (Wang et al., 2026; Xu et al., 2025). Within East Asia the *Mycobacterium avium* complex (MAC) accounts for 85–90% of NTM-PD in Japan and Korea, whereas the *M. abscessus* complex (MABC) reaches 20–30% in southern China and Southeast Asia (Xu et al., 2025; Tan et al., 2018).

Treatment remains unsatisfactory. Standard MAC therapy – a macrolide, ethambutol and a rifamycin – achieves sputum-culture conversion in 50–70% of patients and fails in 25–60% of those with cavitary or advanced disease (Kang et al., 2021; Nguyen et al., 2025; van Ingen et al., 2013). MABC-PD responds in 30–50% of cases even under guideline-recommended regimens (Lim et al., 2025; Cheng et al., 2023; Tunesi et al., 2024), whereas *M. kansasii*-PD exceeds 90% treatment success with rifampicin- and ethambutol-based therapy (Park et al., 2010). Regimens are long and poorly tolerated: a recent European cohort recorded adverse events in three-quarters of patients and permanent discontinuation in one-fifth (Carli et al., 2025).

Susceptibility testing is advocated once an isolate is recovered, but the 2020 ATS/ERS/ESCMID/IDSA guideline restricts actionable *in vitro* testing to the macrolides and amikacin for MAC and identifies the value of testing other agents as an explicit evidence gap (Daley et al., 2020; Lange et al., 2022). This restriction reflects two considerations. Intrinsic resistance in NTM arises from cell-wall impermeability, drug-modifying enzymes, target-site alteration and active efflux (Tursun et al., 2025), so that *in vitro* categories often reflect species-level biology rather than acquired resistance; and for agents other than the macrolides and amikacin, the correlation between MIC and treatment response in MAC-PD is weak, ethambutol, the rifamycins and the fluoroquinolones all contributing clinically at concentrations an assay would classify as high (Daley et al., 2020; Fröberg et al., 2023). Clinically decisive resistance in MAC is dominated by *rrl* (23S rRNA) mutation, which confers class-wide macrolide cross-resistance. Its emergence is suppressed by companion agents (Griffith et al., 2006; Moon et al., 2016). In MABC the corresponding determinants are *erm(41)*-mediated inducible methylation and *rrl* mutation (Nash et al., 2009). Where no validated breakpoint exists, the categories that reach the clinician are defined locally, and their comparability between laboratories and over time has received little attention.

We set out to define species distribution and MIC profiles in a Shandong NTM population, separating agents with validated CLSI clinical breakpoints from those for which only locally defined categories exist; to describe outcomes of susceptibility-informed therapy across the three major NTM-PD phenotypes; and to identify clinical determinants of cure in MAC-PD.

## 2 Materials and methods

### 2.1 Study design and cohorts

Consecutive patients with NTM-PD who underwent species identification and susceptibility testing at Shandong Public Health Clinical Center between 1 February 2021 and 31 October 2024 were enrolled. Diagnosis followed the 2020 ATS/ERS/ESCMID/IDSA criteria (Daley et al., 2020). Isolated extrapulmonary infection without pulmonary involvement and concurrent *M. tuberculosis* complex infection were exclusions. Duplicate isolates from the same patient were removed, so that one index isolate was analyzed per patient; *n* = 606 denotes 606 patients contributing 606 isolates. Microbiological results are reported per isolate and clinical results per patient.

The center is the provincial referral hospital for tuberculosis and other mycobacterial disease in Shandong Province, and many patients return to local facilities for long-term treatment once the diagnostic work-up is complete. Outcome analysis was therefore performed in a per-protocol completer cohort: patients aged ≥18 years with MAC-PD, MABC-PD or *M. kansasii*-PD who received at least 12 months of continuous susceptibility-informed therapy at this center with complete follow-up. Of the 606 patients, 39 with rare species were excluded; of the remaining 567, a further 440 were excluded, leaving 127 (21.0%) for outcome analysis (Figure 1). Eligibility required survival and continued tolerance of prolonged therapy, so the cohort is not an intention-to-treat population and its cure proportions are upper estimates. Baseline characteristics of completers and excluded patients are compared in Supplementary Table 2.

**FIGURE 1:**
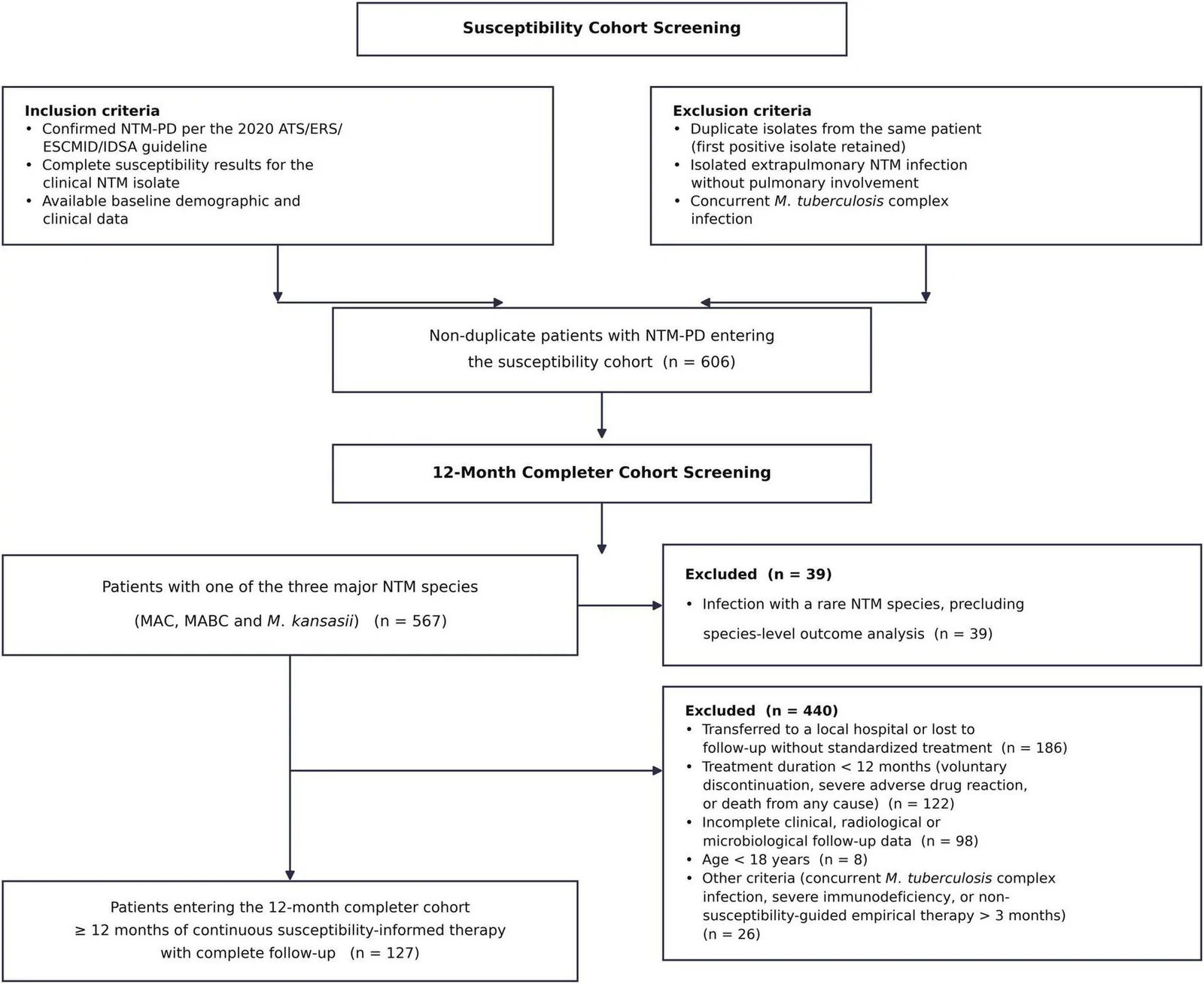
Patient screening and study flow. Enrolment, exclusion and inclusion for the 606-patient susceptibility cohort and the 127-patient 12-month completer cohort, following STROBE reporting guidelines.

### 2.2 Ethics

The study was approved by the Ethics Committee of Shandong Public Health Clinical Center, Shandong University (approval no. GWLCZXEC-SOP-K-2025-103). Written informed consent was waived for this retrospective analysis of de-identified data collected during routine care.

### 2.3 Species identification and susceptibility testing

Species were identified by DNA microarray hybridization (CapitalBio Corporation, Beijing, China) combined with MALDI-TOF mass spectrometry (Microflex LT/SH Biotyper; Bruker Daltonics, Billerica, MA, USA) on BACTEC MGIT 960 culture-positive isolates (Wang et al., 2026); discrepant results were resolved by *hsp65* or *rpoB* sequencing.

Susceptibility to 15 agents was determined by broth microdilution with a commercially prepared dehydrated 96-well panel (Zhuhai Yinke Medical Engineering Co., Ltd., Zhuhai, China; device registration no. 20162400814, Guangdong Medical Products Administration), following the manufacturer’s instructions for use. The procedure follows the broth-microdilution principle described in CLSI M24, 3rd edition (CLSI, 2018a), but the concentration series is that of the commercial panel rather than the doubling-dilution series specified by CLSI; a single panel product was used for the whole study period and its tested concentrations are listed in Supplementary Table 1b. Isolates in logarithmic-phase growth were ground and adjusted to a turbidity of 1 mg/mL, corresponding to approximately 3 × 10^7^–10^8^ CFU/mL. Susceptibility medium and panel were brought to near room temperature before inoculation and the supplied antibacterial supplement was added to the medium to suppress non-mycobacterial growth; 200 μL of inoculated medium was dispensed into each drug well, with sterility controls in wells A1 and B1, growth controls in C1 and D1 and 1/10 and 1/100 growth-control dilutions in E1 and F1. Plates were sealed around the edge with transparent tape and incubated at 37 °C. Plates were first inspected 2 days after inoculation and then every 1–2 days, and the MIC was read once the growth-control wells showed growth, as the lowest concentration completely inhibiting visible growth, judged as the absence of a cell pellet at the bottom of the well; in practice this point was reached on days 3–5 for rapidly growing species and at about day 7 for slowly growing species, extended to days 10–14 where growth was poor. Results below the lowest tested concentration were reported as ≤ that concentration and were assigned a value of half the lowest tested concentration for analysis. An isolate whose control wells remained unchanged beyond day 21 was subcultured and retested. Following CLSI M24, clarithromycin against rapidly growing species was read after extended incubation to day 14 – resistance being reported earlier where the MIC reached ≥ 16 mg/L – so that inducible *erm(41)*-mediated resistance was captured phenotypically. Plates were read independently by two technologists, with discrepancies of more than one dilution adjudicated by a senior microbiologist. Quality-control strains (*M. abscessus* ATCC 19977, *M. avium* ATCC 25291 and *M. kansasii* ATCC 12478) were tested with every run and every new panel lot, and results were accepted only when control MICs fell within the ranges specified in CLSI M24. The assay yields MIC values only; it carries no susceptible, intermediate or resistant criteria for nontuberculous mycobacteria.

*Interpretive criteria*. Susceptibility categories are assigned by the clinical microbiology laboratory, using breakpoints that the laboratory itself defines, and were applied as they stood at the time of testing. The laboratory revised those breakpoints twice during the study period as successive CLSI standards were adopted, defining three periods (Supplementary Table 1a). Before April 2021 it applied in-house criteria derived from early literature and the package insert, stricter than any CLSI edition for the first-line agents (clarithromycin susceptible ≤ 0.5 mg/L; amikacin ≤ 4 mg/L). From April 2021 to May 2024 the second-line criteria were aligned with CLSI M24 (3rd edition) and the interpretive supplement then in force, M62 (CLSI, 2018b), later redesignated M24S – linezolid susceptible ≤ 8 mg/L, moxifloxacin susceptible ≤ 1 and resistant ≥ 4 mg/L – while the first-line criteria were left unchanged pending local verification. From May 2024 the laboratory adopted CLSI M24S, 2nd edition (CLSI, 2023) in full (clarithromycin susceptible ≤ 8, resistant ≥ 32 mg/L; amikacin ≤ 16 and ≥ 64 mg/L) and replaced range-style reporting with explicit ≤ and ≥ criteria. Moxifloxacin was tested against MAC throughout the study period. The interval between publication of a standard and its local implementation – about 2.5 years for M24 (3rd edition) and about 15 months for M24S (2nd edition, published February 2023) – reflects the verification, laboratory-information-system reconfiguration, staff training and quality-control comparison required before clinical deployment.

Three measures limit the effect of these transitions. MIC values are used as the primary susceptibility data throughout (Supplementary Table 3); categorical analyses are stratified by breakpoint period (Supplementary Table 6); and the four MAC agents for which CLSI defines a clinical breakpoint were reclassified directly from individual MIC values against a single criterion – susceptible ≤ 8, intermediate 16, resistant ≥ 32 mg/L for clarithromycin and linezolid; ≤ 16/32/≥ 64 mg/L for amikacin; ≤ 1/2/≥ 4 mg/L for moxifloxacin (CLSI, 2018b) – so that one standard applies across the whole study period. The categories reported at the time of testing and the reclassified categories are compared in Supplementary Table 5. For all other agents, including ethambutol and the rifamycins, no validated clinical breakpoint exists; their results are reported as low, intermediate and high MIC categories and carry no claim of clinical resistance.

The scale of the effect can be stated directly. Seven of the fifteen agents tested against MAC had their category thresholds revised at least once. The tested concentrations did not change – 99.4% of 7,889 recorded MIC values correspond to a tested concentration or to the below-range convention – yet the same MIC value was assigned to different categories at different times in 18 of the 41 species–agent pairs examined (Supplementary Table 1c). Categorical results for agents without a CLSI clinical breakpoint are therefore not comparable between periods.

Denominators are given per agent. Where a category was derived from the MIC, the denominator is the number of isolates with a valid MIC result; where the laboratory-assigned category is reported, it is the number carrying an assigned category. The two differ for two MAC agents: ethambutol (443 MIC results, 442 categories) and tobramycin (442 MIC results, 443 categories).

### 2.4 Treatment and outcome definitions

Patients received individualized multidrug therapy composed of guideline-recommended agents, the final regimen being determined by the attending physician from the susceptibility profile, age and comorbidity, drug–drug interactions and anticipated tolerability (Lange et al., 2022). Doses followed a fixed institutional protocol, several agents being weight-banded at a 50 kg threshold, and are given in Supplementary Table 9c. Rifapentine replaced rifampicin in 15 patients who could not tolerate daily rifampicin; it is not among the rifamycins recommended for MAC-PD.

Core agents (a macrolide, a rifamycin and ethambutol) were distinguished from adjunctive agents (amikacin, fluoroquinolones and linezolid). MAC-PD regimens were classified as standard triple-core, regimen 1 (two core agents plus at least one adjunctive agent in the lowest tested MIC category) or regimen 2 (one core agent plus at least two such adjunctive agents); MABC-PD regimens as linezolid-containing or linezolid-free; and *M. kansasii*-PD regimens as containing or lacking the guideline three-drug core. Regimen group denotes the regimen received for the greater part of the course. Mid-course modification was recorded in 5.9% of the standard triple-core group but in 100% of MAC regimen 2 and 90.0% of the *M. kansasii* group without a guideline core (Supplementary Table 9b), so regimen group is substantially a consequence of events during therapy and is treated as descriptive throughout.

Outcome definitions were adapted from the 2018 NTM-NET consensus statement (van Ingen et al., 2018). Cure required sustained sputum-culture conversion – two consecutive negative cultures obtained at least 4 weeks apart – maintained for at least 12 months on therapy, together with stable or improved radiology; failure denoted persistent culture positivity or clinical or radiographic progression. Induced sputum was used where expectoration was inadequate (Haworth et al., 2017). Because eligibility required 12 months of continuous therapy with complete follow-up, patients who died, discontinued or were lost were excluded at screening rather than censored within the analysis, and every analyzed patient is classified as cure or failure. Microbiological recurrence after conversion was recorded separately. For 19 of the 22 MABC-PD patients the end-of-treatment radiology report could not be retrieved in structured form; radiological stability or improvement had been documented in the clinical record and was used in assigning outcome.

### 2.5 Statistical analysis

Analyses used SPSS 26 (IBM Corp., Armonk, NY, USA) and Python 3.11. Continuous variables were compared with the Mann–Whitney U or Student *t*-test. Every contingency table examined contained at least one expected count below 5, so association *P*-values are Monte-Carlo estimates of the exact *P*-value (20,000 samples; seed 20260906) or Fisher exact tests for 2 × 2 tables, with the Pearson χ^2^ statistic reported descriptively only. Cramér’s V and Cohen’s κ quantified inter-drug association and category agreement. Co-occurrence proportions were calculated on a complete-case basis with combination-specific denominators and no imputation; because the same isolates contribute to many overlapping combinations, combinations were not compared statistically. Completers and excluded patients were compared using standardized mean differences (SMD) alongside significance tests, an absolute SMD below 0.10 indicating negligible imbalance. Subgroups of fewer than 25 patients are described without inferential testing.

Cure in MAC-PD (*n* = 85; 50 cured, 35 failures) was modeled by binary logistic regression with forced entry of three pre-specified covariates: radiographic pattern, regimen category and total drug count. With six degrees of freedom the events-per-variable ratio was 5.8, and the model is treated as exploratory. Robustness was assessed with a parsimonious model containing radiographic pattern alone and with a Firth penalized-likelihood model (Heinze and Schemper, 2002); full output is given in Supplementary Table 7. Variance inflation factors did not exceed 5. Model fit was assessed by the omnibus test, the Hosmer–Lemeshow statistic and Nagelkerke R^2^. Tests were two-sided at α = 0.05.

## 3 Results

### 3.1 Species distribution and patient demographics

MAC accounted for 73.1% of the 606 isolates, MABC for 12.4% and *M. kansasii* for 8.1%; *M. fortuitum*, *M. chelonae* and other rare species made up the remainder (Figure 2). This profile matches contemporary mainland-China surveillance and differs from the MABC-rich pattern of southern China (Xu et al., 2025; Tan et al., 2018).

**FIGURE 2:**
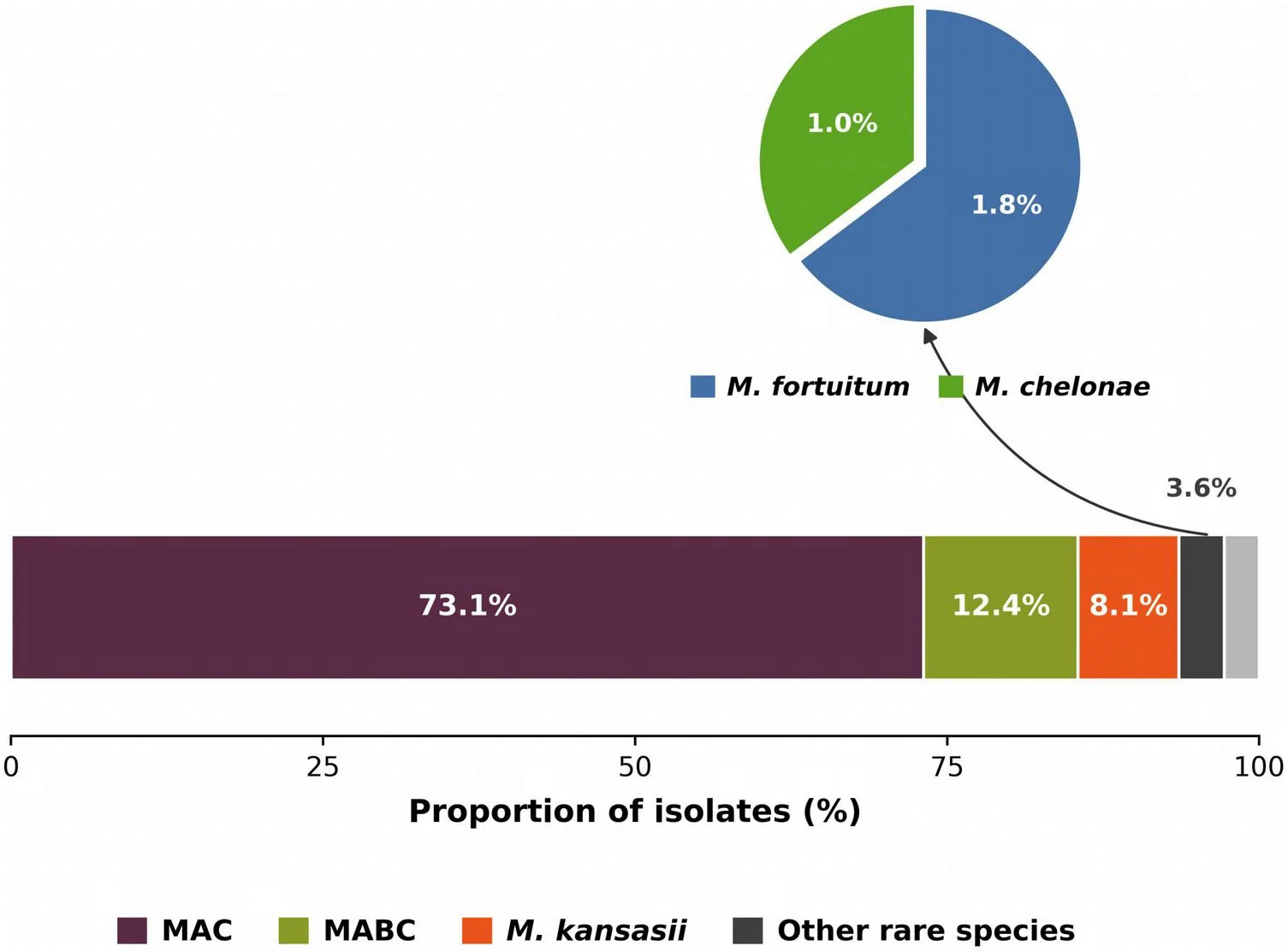
Species distribution of 606 NTM clinical isolates, one per patient. The inset resolves the two named rare species, *M. fortuitum* (1.8%) and *M. chelonae* (1.0%); the remaining 3.6% comprises other rare species.

Demography was strongly species-dependent (Figure 3). The cohort as a whole was 54.8% male with a mean age of 58.6 ± 14.5 years, but MAC and *M. kansasii* were male-predominant (52.6% and 89.8% male) whereas MABC was predominantly female (64.0%) and affected younger patients (mean 51.5 ± 18.1 years) than MAC (61.6 ± 12.5 years). *M. kansasii* patients were younger still (45.0 ± 12.7 years). Among the 263 women with MAC, MABC or *M. kansasii* disease the age distribution showed two local maxima, at 50–54 and 65–69 years (Supplementary Figure 1).

**FIGURE 3:**
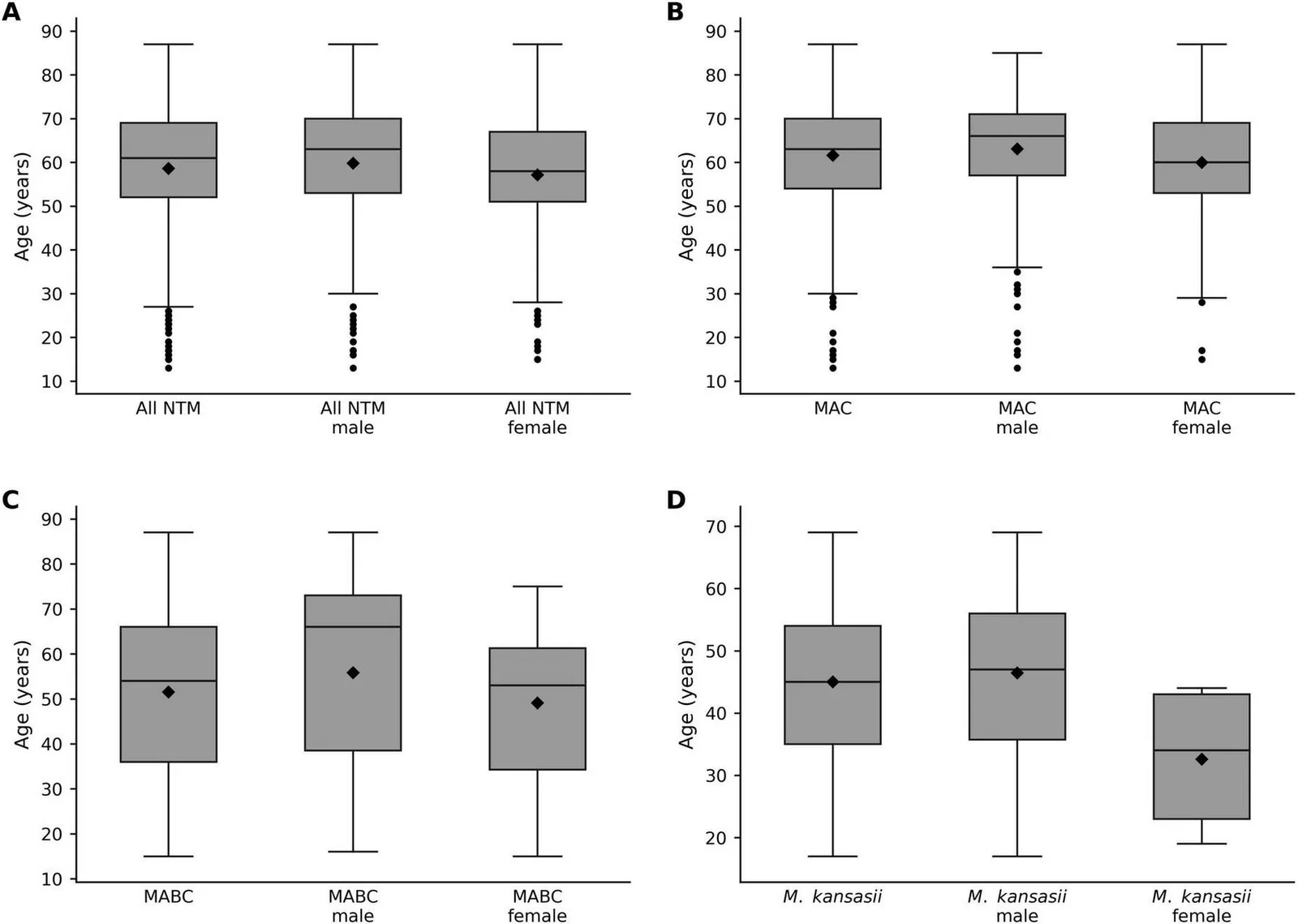
Sex-stratified age distribution of NTM-PD patients. **(A)** Whole cohort; **(B–D)** MAC, MABC and *M. kansasii*. Box, interquartile range; central line, median; whiskers, 1.5 × IQR; diamond, mean; dots, outliers. *M. fortuitum* and other rare species were excluded (*n* < 20 per subgroup). A histogram of female age in 5-year bins is given as Supplementary Figure 1.

### 3.2 Antimicrobial MIC profiles

Full MIC distributions are given in Supplementary Table 3 and categorical summaries in Table 1. Applied directly to individual MIC values, the CLSI clinical breakpoints show near-complete *in vitro* activity of all four validated agents against MAC: 98.9% of isolates were susceptible to clarithromycin, 99.8% to amikacin, 99.3% to linezolid and 96.2% to moxifloxacin. No MAC isolate had a moxifloxacin MIC above 4 mg/L although the dilution series extends to 16 mg/L, so the high susceptibility is not an artifact of a truncated range. Categories as reported at the time of testing and categories reclassified against the CLSI criterion diverged most for amikacin and linezolid, whose earlier local thresholds placed fully susceptible isolates in an intermediate category (Supplementary Table 5).

**TABLE 1:** MIC-category profiles of 15 agents against NTM isolates, given as n (low/intermediate/high) and percentage.

Agent	MAC (*n* = 443)	MABC (*n* = 75)	*M. kansasii* (*n* = 49)	*M. *fortuitum* (*n* = 11)*
RIF	440/1/2 (99.3/0.2/0.5)	–	47/0/2 (95.9/0/4.1)	–
CLA	438/1/4 (98.9/0.2/0.9)	67/7/1 (89.3/9.3/1.3)	49/0/0 (100/0/0)	9/2/0 (81.8/18.2/0)
RFB	438/1/4 (98.9/0.2/0.9)	–	47/0/2 (95.9/0/4.1)	–
AMK	442/0/1 (99.8/0/0.2)	75/0/0 (100/0/0)	49/0/0 (100/0/0)	10/0/1 (90.9/0/9.1)
LZD	440/1/2 (99.3/0.2/0.5)	74/0/1 (98.7/0/1.3)	49/0/0 (100/0/0)	11/0/0 (100/0/0)
MXF	426/15/2 (96.2/3.4/0.5)	62/9/4 (82.7/12.0/5.3)	48/0/1 (98.0/0/2.0)	10/0/1 (90.9/0/9.1)
FOX	418/13/12 (94.4/2.9/2.7)	65/9/1 (86.7/12.0/1.3)	–	11/0/0 (100/0/0)
TOB	181/242/20 (40.9/54.6/4.5)	3/15/57 (4.0/20.0/76.0)	–	1/2/8 (9.1/18.2/72.7)
AZM	136/210/97 (30.7/47.4/21.9)	30/17/11 (51.7/29.3/19.0; *n* = 58)	–	1/0/6 (14.3/0/85.7; *n* = 7)
GAT	99/162/182 (22.3/36.6/41.1)	–	37/0/2 (94.9/0/5.1; *n* = 39)	–
EMB	73/172/197 (16.5/38.9/44.6; *n* = 442)	–	39/3/7 (79.6/6.1/14.3)	–
SXT	74/14/355 (16.7/3.2/80.1)	19/4/51 (25.7/5.4/68.9; *n* = 74)	13/1/35 (26.5/2.0/71.4)	4/0/7 (36.4/0/63.6)
IMP	40/3/400 (9.0/0.7/90.3)	1/1/73 (1.3/1.3/97.3)	–	1/0/10 (9.1/0/90.9)
DOX	9/8/426 (2.0/1.8/96.2)	6/3/66 (8.0/4.0/88.0)	–	1/0/10 (9.1/0/90.9)
MIN	8/9/426 (1.8/2.0/96.2)	8/2/48 (13.8/3.4/82.8; *n* = 58)	–	0/2/5 (0/28.6/71.4; *n* = 7)

For MAC, the four agents with CLSI clinical breakpoints (CLA, AMK, LZD, MXF) are categorized directly from individual MIC values – susceptible ≤ 8/intermediate 16/resistant ≥ 32 mg/L for clarithromycin and linezolid, ≤ 16/32/ ≥ 64 mg/L for amikacin and ≤ 1/2/ ≥ 4 mg/L for moxifloxacin – and their labels correspond to susceptible/intermediate/resistant. For MABC and *M. kansasii* the reported categories were checked against the same direct recalculation: it reproduced them exactly for clarithromycin, amikacin, linezolid, moxifloxacin, cefoxitin, doxycycline and minocycline in MABC, and for clarithromycin, rifampicin, amikacin, linezolid and moxifloxacin in *M. kansasii*, so those entries are CLSI-consistent (Supplementary Table 5). Imipenem and tobramycin in MABC, ethambutol and rifabutin in *M. kansasii*, and trimethoprim–sulfamethoxazole in every species are reported as assigned by the laboratory, recalculation having been either discordant or, for trimethoprim–sulfamethoxazole, impossible because only the sulfamethoxazole component is recorded. For all remaining agents, including ethambutol and the rifamycins, no validated clinical breakpoint exists; the categories are those assigned by the laboratory using its own breakpoints and denote MIC distribution only. Because those breakpoints were revised twice during the study period as successive CLSI standards were adopted (Supplementary Table 1a), these categories are not comparable between periods. Full MIC distributions are given in Supplementary Table 3, and the categories as reported at the time of testing are compared with the CLSI-recalculated categories in Supplementary Table 5. A dash indicates that the agent was not routinely tested against that species. Where an agent was tested in fewer isolates than the species total, or where an isolate carried an MIC without an assigned category, the denominator used is printed in the cell; all other cells use the species total in the column heading. Ethambutol in MAC has 443 MIC results and 442 assigned categories, and the categorical denominator is used here. For MABC, clarithromycin was read at day 14 after extended incubation; subspecies were not differentiated. RIF, rifampicin; CLA, clarithromycin; RFB, rifabutin; AMK, amikacin; LZD, linezolid; MXF, moxifloxacin; FOX, cefoxitin; TOB, tobramycin; AZM, azithromycin; GAT, gatifloxacin; EMB, ethambutol; SXT, trimethoprim–sulfamethoxazole; IMP, imipenem; DOX, doxycycline; MIN, minocycline.

Ethambutol behaved differently. Its MIC distribution was displaced toward the upper end of the four tested concentrations, with 16.5% of isolates inhibited below 2.5 mg/L and 44.6% at 5 mg/L or above (MIC_50_ 2.5 mg/L, MIC_90_ 10 mg/L). Because no clinical breakpoint exists for ethambutol against MAC, these values describe the distribution and not resistance. Analysis by year of report showed no monotone trend (Spearman ρ = −0.088, *P* = 0.063). Distributions differed between years (Kruskal–Wallis H = 118.2, *P* < 0.001), but the difference was confined to 2023 – 80.6% in the highest category against 24.1–39.3% in every other year – and the 2024 distribution reverted to the earlier pattern (Supplementary Table 4). Neither the ethambutol criteria nor the tested concentrations changed in that year, and the records available to us do not identify a methodological or case-mix explanation for the excursion.

The dependence of categorical output on the criteria in force was quantified directly. Comparing breakpoint periods, 6 of 11 comparisons differed significantly among agents without a CLSI clinical breakpoint, against 2 of 21 among agents with one (Supplementary Table 6). Azithromycin fell into higher categories far more often than clarithromycin (Table 1), a difference generated by the thresholds applied to the two macrolides rather than by independent resistance. Imipenem and the tetracyclines showed uniformly high MICs against MAC.

Against MABC, amikacin and linezolid were almost uniformly low-MIC (100% and 98.7%), clarithromycin 89.3% at the day-14 reading and moxifloxacin 82.7%, while imipenem, tobramycin and the tetracyclines were predominantly high. *M. kansasii* was uniformly low-MIC to clarithromycin, amikacin and linezolid and 95.9% low-MIC to both rifamycins.

### 3.3 Co-occurrence of low MIC categories in MAC and MABC

Isolates simultaneously occupying the lowest MIC category for every constituent agent were enumerated for all three- to six-agent combinations of 11 agents per species (Figures 4, 5; complete listing in Supplementary Table 8). This is a co-occurrence calculation on single-agent data, and no interaction assay was performed. In MAC the leading combinations paired a rifamycin and clarithromycin with amikacin, moxifloxacin or linezolid, exceeding 94% for three agents and remaining above 85% for six-agent combinations that excluded tobramycin; including tobramycin reduced the best six-agent proportion from 89.8% to 39.7%. In MABC the highest-ranking combination was linezolid with clarithromycin and amikacin (89.3%), and proportions fell steeply as further agents were added. Ethambutol was excluded from the MAC analysis because it is retained in the regimen irrespective of its MIC.

**FIGURE 4:**
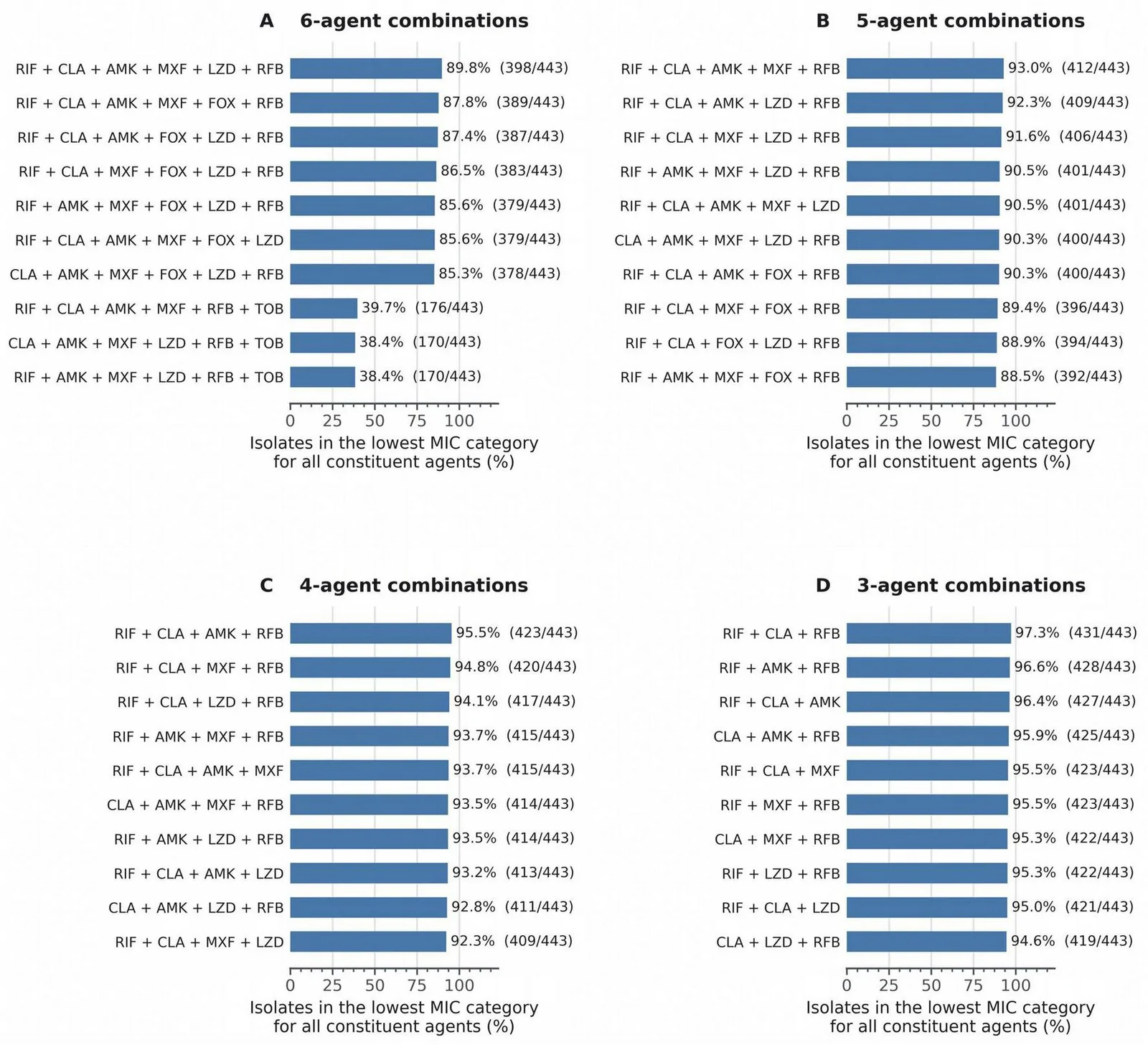
Co-occurrence of low MIC categories in MAC isolates, for combinations of six **(A)**, five **(B)**, four **(C)** and three **(D)** agents. Bars show the percentage of tested isolates falling simultaneously in the lowest MIC category for every constituent agent, with the count and complete-case denominator beside each bar; the ten highest-ranking combinations are shown per panel and the complete listing is in Supplementary Table 8. This is a co-occurrence calculation on single-agent MIC data; no antimicrobial interaction was assessed. Denominators are complete-case and differ between combinations, which are therefore not compared statistically. For MAC every constituent agent has *n* = 443. RIF, rifampicin; CLA, clarithromycin; AMK, amikacin; MXF, moxifloxacin; FOX, cefoxitin; LZD, linezolid; RFB, rifabutin; GAT, gatifloxacin; SXT, trimethoprim–sulfamethoxazole; AZM, azithromycin; TOB, tobramycin.

**FIGURE 5:**
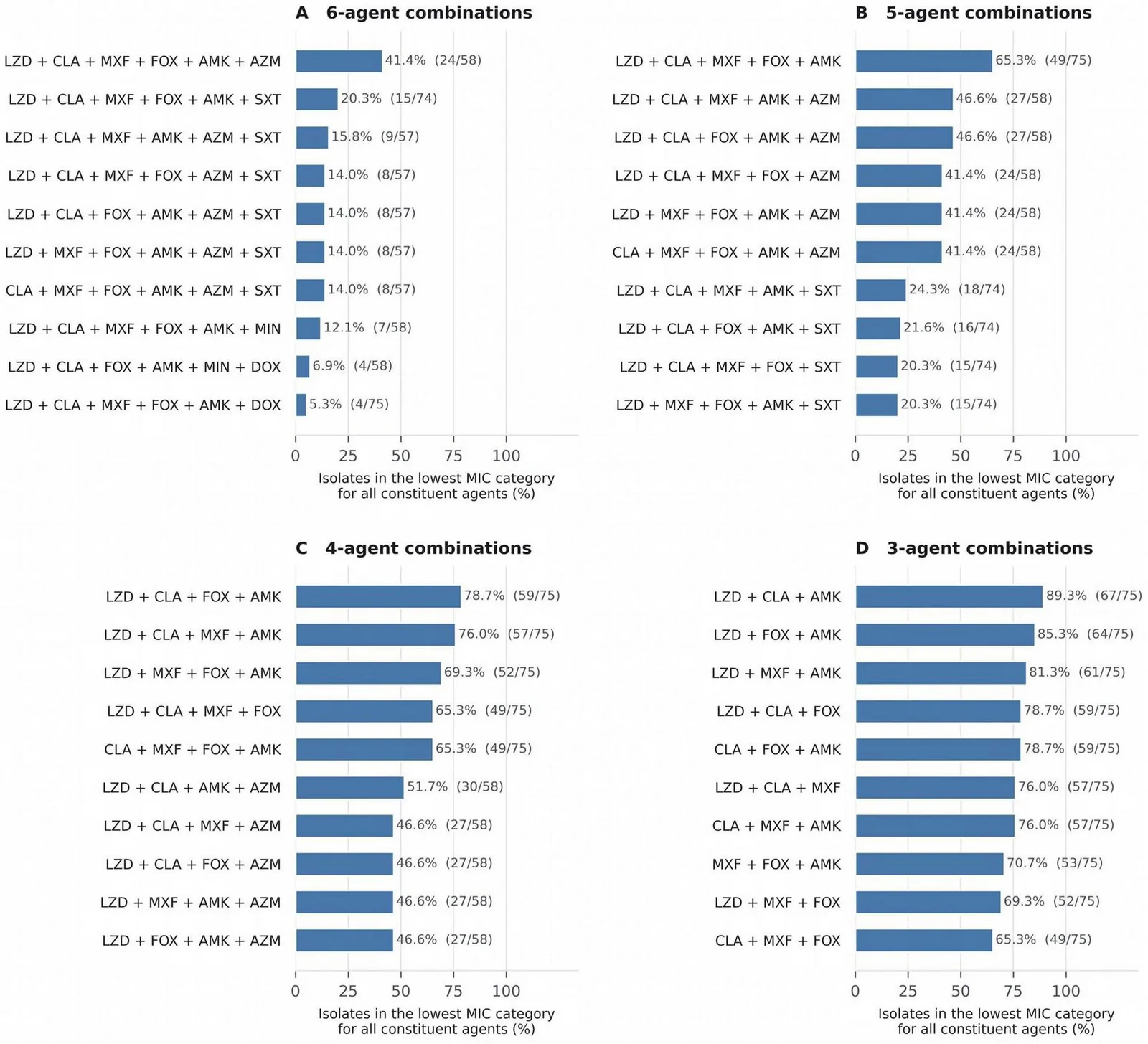
Co-occurrence of low MIC categories in MABC isolates, for combinations of six **(A)**, five **(B)**, four **(C)** and three **(D)** agents, presented as in Figure 4. Per-agent denominators are 58 for azithromycin and minocycline, 74 for trimethoprim–sulfamethoxazole and 75 for the remaining agents; complete-case denominators for combinations therefore range from 57 to 75 and are printed beside each bar. DOX, doxycycline; MIN, minocycline.

### 3.4 Association among congeneric agents

Within-class concordance was assessed for four congeneric pairs in MAC and three in MABC (Tables 2, 3). Clarithromycin and azithromycin categories were discordant (Cramér’s V = 0.153, κ = 0.011), with 93 of 97 azithromycin high-MIC isolates remaining in the lowest clarithromycin MIC category; this follows from the different thresholds applied to the two macrolides rather than from separate resistance mechanisms. Rifampicin and rifabutin were moderately concordant (V = 0.247, κ = 0.245), and amikacin MICs were consistently lower than tobramycin MICs. In MABC, minocycline and doxycycline were closely concordant (V = 0.655, κ = 0.604), so testing one tetracycline approximates the other.

**TABLE 2:** Cross-classification and association of MIC categories among congeneric agents in MAC isolates (*n* = 443).

Category of reference agent	AZM-S	AZM-I	AZM-R
CLA / AZM – Pearson χ2 = 10.383 (descriptive), df = 2; Monte-Carlo exact P = 0.007; Cramér’s V = 0.153; κ = 0.011			
CLA-S	135	210	93
CLA-I	0	0	0
CLA-R	1	0	4
**Category of reference agent**	**RFB-S**	**RFB-I**	**RFB-R**
RIF / RFB – Pearson χ2 = 54.133 (descriptive), df = 4; Monte-Carlo exact P = 0.033; Cramér’s V = 0.247; κ = 0.245			
RIF-S	436	1	3
RIF-I	1	0	0
RIF-R	1	0	1
**Category of reference agent**	**TOB-S**	**TOB-I**	**TOB-R**
AMK / TOB – Pearson χ2 = 7.087 (descriptive), df = 4; Monte-Carlo exact P = 0.099; Cramér’s V = 0.089; κ = 0.017			
AMK-S	180	236	19
AMK-I	0	6	1
AMK-R	1	0	0
**Category of reference agent**	**GAT-S**	**GAT-I**	**GAT-R**
MXF / GAT – Pearson χ2 = 20.732 (descriptive), df = 2; Monte-Carlo exact P < 0.001; Cramér’s V = 0.216; κ = −0.006			
MXF-S	99	162	168
MXF-I	0	0	14
MXF-R	0	0	0

Rows are the reference agent, columns the comparator. Every table contained at least one expected count below 5, so P values are Monte-Carlo estimates of the exact P value (20,000 samples; seed 20260906) and the Pearson χ^2^ statistic is descriptive. Azithromycin and clarithromycin use different category thresholds, so the association statistics describe category concordance only. Every category in this table – for clarithromycin, amikacin and moxifloxacin as well as for their comparators – is the category as reported at the time of testing, so that each congeneric pair is compared on a single basis; the comparator agents (azithromycin, rifabutin, tobramycin and gatifloxacin) have no CLSI clinical breakpoint and no reclassified category exists for them. Row margins here therefore differ from Table 1, which reports CLSI-reclassified categories for the four agents with clinical breakpoints: clarithromycin 438/0/5 here against 438/1/4 in Table 1, and amikacin 435/7/1 here against 442/0/1 in Table 1. The reclassified values are given in Table 1 and Supplementary Table 5.

**TABLE 3:** Cross-classification and association of MIC categories among congeneric agents in MABC isolates.

Category of reference agent	AZM-S	AZM-I	AZM-R
CLA / AZM (n = 58) – Pearson χ2 = 13.740 (descriptive), df = 2; Monte-Carlo exact P = 0.002; Cramér’s V = 0.487; κ = 0.073			
CLA-S	30	16	7
CLA-I	0	1	4
CLA-R	0	0	0
**Category of reference agent**	**DOX-S**	**DOX-I**	**DOX-R**
MIN / DOX (n = 58) – Pearson χ2 = 49.755 (descriptive), df = 4; Monte-Carlo exact P < 0.001; Cramér’s V = 0.655; κ = 0.604			
MIN-S	4	3	1
MIN-I	0	0	2
MIN-R	0	0	48
**Category of reference agent**	**TOB-S**	**TOB-I**	**TOB-R**
AMK / TOB (n = 75) – not calculable (single non-empty row); κ = 0.000			
AMK-S	3	15	57
AMK-R	0	0	0

Layout, tests and category provenance as in Table 2: all categories are those reported at the time of testing. Azithromycin and minocycline were tested in 58 isolates, amikacin and tobramycin in all 75.

### 3.5 Slow-growing versus rapidly growing mycobacteria

Linezolid, amikacin, clarithromycin, cefoxitin and moxifloxacin showed low MICs across the rapidly growing mycobacteria. MABC and *M. fortuitum* showed elevated tobramycin and imipenem MICs, consistent with the intrinsic-resistance mechanisms described for this group (Tursun et al., 2025). Among the slowly growing species, MAC and *M. kansasii* were separated principally by the rifamycins and ethambutol, for which *M. kansasii* MICs were uniformly low.

### 3.6 Clinical outcomes in the 12-month completer cohort

Completers differed from excluded patients principally in age (54.6 ± 14.6 vs. 60.0 ± 14.2 years, *P* < 0.001; SMD −0.38). The proportion with any cavitation was similar in the two groups (59.8% vs. 56.8%, *P* = 0.544; SMD + 0.06) and the distribution of radiographic patterns did not differ (*P* = 0.786), indicating that selection into the cohort did not operate through the principal prognostic variable (Supplementary Table 2).

Cure was achieved in 58.8% of MAC-PD patients (50/85), 54.5% of MABC-PD patients (12/22) and 95.0% of *M. kansasii*-PD patients (19/20). Because the outcomes of excluded patients are unknown, assuming failure in all of them bounds these proportions at 11.3%, 16.0% and 38.8% respectively. Microbiological recurrence after conversion occurred in 9 of the 81 cured patients (11.1%) and was most frequent in MABC-PD (4/12).

Median duration of therapy was 16.0 months in MAC-PD, 18.0 months in MABC-PD and 14.0 months in *M. kansasii*-PD, with median culture conversion at 3.7, 3.6 and 2.7 months (Supplementary Table 9a). Intravenous amikacin was given to 14.1% of MAC-PD and 36.4% of MABC-PD patients, and linezolid was included in 24.7% and 50.0% of their regimens. The regimen was modified during treatment in 36.2% of patients, most often for gastrointestinal intolerance or hematological toxicity, with rifampicin the agent most frequently implicated (Supplementary Tables 9d,e).

In MAC-PD, cure fell from 79.4% in patients with a bronchiectasis-nodular pattern to 50.0% with mixed disease and 40.0% with cavitary disease. In the multivariable model (omnibus χ^2^ = 24.331, df = 6, *P* < 0.001; Hosmer–Lemeshow *P* = 0.137; Nagelkerke R^2^ = 0.335), cavitation was associated with substantially lower odds of cure (adjusted odds ratio 0.146, 95% CI 0.040–0.538, *P* = 0.004), with closely similar estimates in the parsimonious (0.173) and Firth penalized-likelihood (0.176) models (Figure 6 and Table 4, and Supplementary Table 7). Regimen category was not associated with cure. Total drug count showed an inverse association (four drugs, adjusted odds ratio 0.186; five or more drugs, 0.081) that is collinear with regimen category by construction and is interpreted as a marker of disease severity.

**FIGURE 6:**
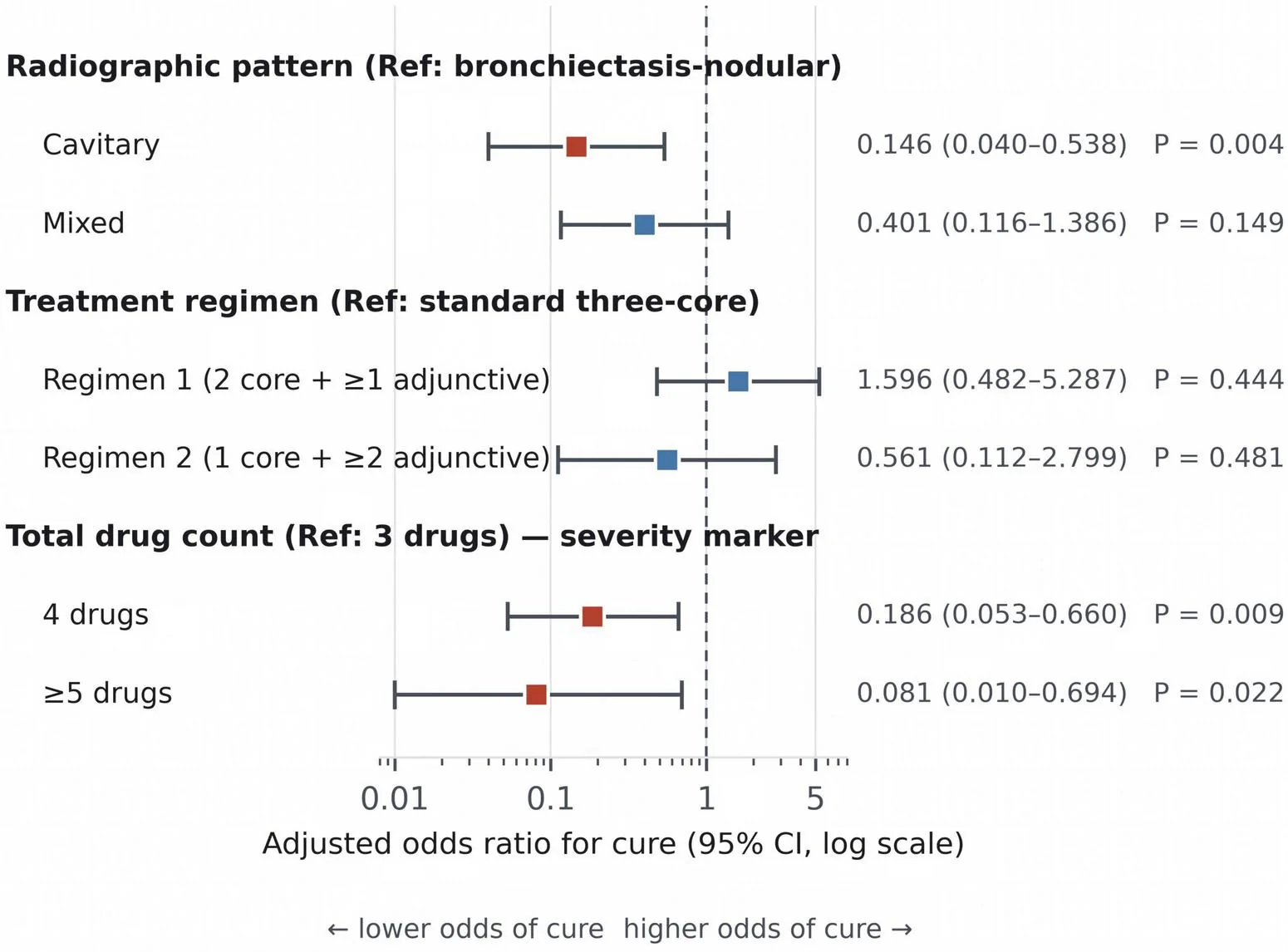
Multivariable logistic regression of cure in MAC-PD among 12-month completers (*n* = 85). Adjusted odds ratios with 95% confidence intervals on a logarithmic scale; red squares indicate *P* < 0.05. Adjusted for radiographic pattern, treatment regimen and total drug count (omnibus χ^2^ = 24.331, df = 6, *P* < 0.001; Hosmer–Lemeshow *P* = 0.137). The regimen and drug-count terms are collinear by construction.

**TABLE 4:** Binary logistic regression of factors associated with cure among MAC-PD 12-month completers (*n* = 85; 50 cured, 35 failures; events per variable 5.8).

Variable	B	SE	Wald χ^2^	df	*P*	aOR	95% CI
Radiographic pattern (Ref: bronchiectasis-nodular)							
Cavitary	−1.924	0.665	8.373	1	0.004	0.146	0.040–0.538
Mixed	−0.914	0.633	2.086	1	0.149	0.401	0.116–1.386
Treatment regimen (Ref: standard triple-core)							
Regimen 1 (2 core + ≥ 1 low-MIC adjunctive)	+0.467	0.611	0.585	1	0.444	1.596	0.482–5.287
Regimen 2 (1 core + ≥ 2 low-MIC adjunctive)	−0.578	0.820	0.497	1	0.481	0.561	0.112–2.799
Total drug count (Ref: 3 drugs) – severity marker							
4 drugs	−1.681	0.646	6.772	1	0.009	0.186	0.053–0.660
≥5 drugs	−2.508	1.093	5.263	1	0.022	0.081	0.010–0.694

B, coefficient; SE, standard error; aOR, adjusted odds ratio. An aOR below 1 indicates lower odds of cure relative to the reference. The regimen and drug-count terms are collinear by construction and total drug count is interpreted as a severity marker. Model fit: omnibus χ^2^ = 24.331, df = 6, *P* < 0.001; Hosmer–Lemeshow χ^2^ = 12.340, df = 8, *P* = 0.137; Nagelkerke R^2^ = 0.335; overall accuracy 67.1%. Parsimonious and Firth penalized-likelihood models are given in Supplementary Table 7.

Ethambutol was withheld because of an elevated MIC in 9 of the 85 MAC-PD patients and was flagged but retained in a further 13 (Supplementary Table 9f), so an *in vitro* category with no validated clinical meaning was already influencing prescribing at this center.

No MABC-PD patient received a strict guideline-recommended intravenous induction phase: cefoxitin is classified as a restricted-use and imipenem as a special-use antimicrobial under this institution’s antimicrobial stewardship policy, and 97.3% of MABC isolates fell in the highest imipenem MIC category. Cure was equally frequent in linezolid-containing and linezolid-free regimens (6/11 in each). In *M. kansasii*-PD, all 10 patients receiving the guideline three-drug core were cured, as were 9 of the 10 whose regimen lacked a core agent – in nine of whom a core drug had been discontinued mid-course for toxicity.

## 4 Discussion

Three findings emerge from this cohort. Against MAC, the agents for which CLSI defines validated clinical breakpoints retain near-complete *in vitro* activity, so categorical susceptibility testing is unlikely to alter the choice of macrolide or aminoglycoside. For the remaining agents no validated breakpoint exists, and the categories that reach the clinician rest on criteria the laboratory defines for itself – criteria that were revised twice during the study period as successive CLSI standards were adopted, with measurable consequences for the categorical output. Finally, among patients who completed at least 12 months of therapy, cure was associated with disease phenotype rather than with regimen composition.

### 4.1 Species distribution and demographics

MAC accounted for nearly three-quarters of isolates, consistent with contemporary Chinese surveillance (Xu et al., 2025), and the contrast with the higher MABC proportion of southern China supports a north–south gradient in NTM ecology within China (Tan et al., 2018). The female predominance of MABC and the male predominance of MAC and *M. kansasii* reproduce patterns described in Japanese and Korean cohorts and in international reviews (Ko et al., 2018; Prevots and Marras, 2015). The age distribution among women showed two local maxima, at 50–54 and 65–69 years; this is descriptive and was not tested formally.

### 4.2 MIC profiles, macrolide categories and the limits of *in vitro* interpretation

Clarithromycin, amikacin and linezolid retained near-complete *in vitro* activity against MAC, whereas moxifloxacin susceptibility changed only marginally, and in the direction opposite to that predicted from the earlier, wider local threshold. Direct reclassification against the CLSI criterion gave 96.2% susceptibility, against 96.8% as originally reported. The local susceptible threshold was ≤ 4 mg/L only under the earliest criteria and ≤ 1 mg/L thereafter, and almost all isolates had MICs at or below 1 mg/L under either rule. Both the susceptible–intermediate and the intermediate–resistant boundaries fell within the tested dilution range, so this level of susceptibility reflects the population rather than the assay.

The apparent clarithromycin–azithromycin discordance is a threshold effect rather than a biological one. In MAC, clinically relevant macrolide resistance is dominated by *rrl* (23S rRNA) mutation and is class-wide, so clarithromycin remains the appropriate class representative for testing and azithromycin should not be regarded as separately informative. In MABC the picture is genuinely different, since inducible *erm(41)* methylation determines macrolide efficacy independently of the *rrl* genotype (Nash et al., 2009), which is why extended incubation to day 14 is essential and why subspecies assignment carries prognostic weight.

Ethambutol illustrates the limits of the categorical output most clearly. Only 16.5% of MAC isolates occupied the lowest ethambutol MIC category while 44.6% occupied the highest, a distribution consistent with that reported by independent Chinese laboratories (Tan et al., 2024). Mechanisms analogous to the *embCAB*-operon alterations that confer high-level ethambutol resistance in *M. tuberculosis* have been described in MAC (Li et al., 2025), but two constraints apply to the interpretation of these categories. No CLSI clinical breakpoint exists for ethambutol against MAC, so the categories are locally defined bins that have never been validated against outcome; and for agents other than the macrolides and amikacin, *in vitro* MICs correlate poorly with treatment response in MAC-PD, ethambutol, the rifamycins and the fluoroquinolones all being active clinically at concentrations that the MIC would classify as high (Daley et al., 2020; Fröberg et al., 2023). We therefore report the ethambutol result as a description of the MIC distribution in this cohort and draw no inference about resistance from it.

The inference that follows rests on pharmacological and genomic evidence rather than on the present cohort. The pharmacological consequence is a safety concern. Ethambutol inhibits arabinosyltransferase and disrupts arabinogalactan synthesis, increasing cell-wall permeability to companion agents; its contribution to the MAC regimen is largely to protect the macrolide rather than to sterilize independently. Macrolide monotherapy, or effective monotherapy created by withdrawing companion drugs, selects *rrl* mutants, which confer high-level class-wide macrolide resistance and are associated with sharply reduced conversion and increased mortality (Griffith et al., 2006; Moon et al., 2016). Withdrawing ethambutol on the basis of an unvalidated category therefore trades an uninterpretable *in vitro* signal for a well-characterized clinical hazard. That 9 of 85 patients in this cohort had ethambutol withheld for precisely this reason indicates that the hazard is not hypothetical.

### 4.3 MAC-PD treatment outcomes

Cavitary disease was associated with substantially lower odds of cure, with an adjusted odds ratio of 0.15 and closely similar estimates across the primary, parsimonious and penalized-likelihood specifications. This accords with evidence that cavity size and lobar extent predict failure more precisely than binary cavitary status (Kang et al., 2021; Kim et al., 2024; Hayashi et al., 2025). The association, if confirmed in prospective cohorts, would justify closer monitoring and earlier consideration of intensification or surgical referral in cavitary disease rather than any modification of drug selection.

Two interpretive cautions follow from the data structure. The events-per-variable ratio of 5.8 is below the conventional threshold of 10, and the wide confidence intervals on the regimen and drug-count terms are a direct expression of that sparseness; only the cavitary association should be regarded as reliable. The inverse association between total drug count and cure is most plausibly explained by confounding by indication (Kyriacou and Lewis, 2016): broader regimens are prescribed for more extensive disease, and severity drives both the regimen and the outcome, as severity-stratified cohorts show (Kim et al., 2022; Fukushima et al., 2020). Drug count is therefore a severity marker, and the absence of any association between regimen category and cure carries no implication that regimens can safely be narrowed. Whether susceptibility testing can guide regimen individualization requires prospective trials with pre-specified margins (Nguyen et al., 2025).

### 4.4 MABC-PD

MABC-PD remains the most difficult phenotype. Cure reached 54.5% although no patient received a strict intravenous induction phase. This lies within the 29–77% range of sputum-culture conversion reported in a recent systematic review (Lim et al., 2025), although the endpoint applied here is stricter and the two are not directly comparable. Three qualifications apply. The subgroup is small (*n* = 22) and selected. Recurrence after conversion was highest in this group (4 of 12 cured patients), so durable success is lower still. MABC subspecies and *erm(41)* sequevar were not determined. Macrolide efficacy differs markedly between subsp. *abscessus*, which carries a functional inducible *erm(41)* allele, and subsp. *massiliense*, in which the allele is truncated and the macrolide remains fully active (Nash et al., 2009). An unmeasured preponderance of subsp. massiliense could therefore account for the observed cure rate without any contribution from the regimen. Cure was equally frequent with and without linezolid, so the uniformly low linezolid MICs provide an *in vitro* rationale (Cheng et al., 2023) that the outcome data neither support nor, at this sample size, could refute. Bedaquiline and clofazimine remain the principal salvage options (Yao et al., 2025).

### 4.5 *M. kansasii*-PD

*M. kansasii*-PD achieved 95.0% cure with no recurrence, consistent with its position as the most treatable of the common NTM species (Park et al., 2010). Near-uniform low MICs to the rifamycins and macrolides underlie this outcome. That 9 of the 10 patients whose regimen lacked a guideline core agent were nonetheless cured – in nine of whom a core drug had been stopped mid-course for toxicity – suggests that the favorable prognosis of this species is robust to regimen attrition, although the sample (*n* = 20) permits no inference.

### 4.6 Congeneric-agent associations

The systematic assessment of within-class concordance answers a practical question and reinforces the interpretive caution above. In MAC, clarithromycin and azithromycin categories were discordant for the threshold reasons already given, and clarithromycin should be the class representative. Rifampicin and rifabutin were moderately but incompletely concordant; since neither has a validated MAC breakpoint, that association describes MIC distributions only. Amikacin MICs were consistently lower than tobramycin MICs, so tobramycin provides little additional information beyond amikacin in MAC. In MABC, minocycline and doxycycline were closely concordant, so testing one tetracycline reasonably approximates the other for descriptive purposes.

### 4.7 Limitations

The outcome cohort is a per-protocol completer cohort. Eligibility required survival and tolerance of at least 12 months of therapy, so patients who died or discontinued early are absent and the cure proportions are upper estimates; the bounding analysis expresses the width of that uncertainty. Completers and excluded patients were closely matched on cavitation, which limits one specific route by which the prognostic model could have been distorted, but unmeasured determinants of completion – comorbidity, disease extent, bacillary load, nutritional status – were not captured. An intention-to-treat cohort could not be assembled because treatment and mortality records for the 186 patients referred back to local hospitals are held by those institutions.

Categorical results for agents without a CLSI clinical breakpoint depend on the criteria in force at the time of testing and are not compared between periods; the MIC distributions are unaffected and are presented as the primary data. All statements about ethambutol and the rifamycins describe MIC distributions rather than validated resistance.

The MAC-PD model is exploratory. The events-per-variable ratio was 5.8, the regimen and drug-count terms are collinear by construction, and there was no concurrent empirically treated comparison group, so no causal comparison of susceptibility-informed against empirical therapy is possible.

Molecular characterization was not performed: *rrl* and *erm(41)* genotypes were not determined and MABC subspecies were not assigned, which limits interpretation of the macrolide results in that species. Radiographic pattern was recorded categorically without quantitative cavity or lobar measurement (Kim et al., 2024; Hayashi et al., 2025).

Exposure data are retrospective. Doses are reported as protocol-level rather than patient-level exposures; adverse events were abstracted from free-text notes and are minimum estimates that are not comparable with prospectively ascertained rates (Carli et al., 2025); and because sputum sampling followed routine care rather than a fixed schedule, times to conversion are interval-censored and approximate. The study is exploratory throughout, with no pre-specified sample size or power calculation, so null findings should not be read as evidence of equivalence.

Three further limits apply. MIC values below the lowest tested concentration were assigned a value of half that concentration for analysis rather than treated as censored observations, which compresses the lower tail of the reported distributions. The 2023 ethambutol excursion described in Section 3.2 was not explained by any change in criteria, tested concentrations or case mix that we could identify. And the study was conducted at a single center using a single assay panel, so the results may not extend to laboratories using different methods or to species other than the four that predominated here.

### 4.8 Clinical and surveillance implications

Three implications follow. First, *in vitro* testing is most actionable for the agents with validated MAC breakpoints and a demonstrated MIC–outcome correlation – the macrolides and amikacin – and clarithromycin should serve as the macrolide class representative. Linezolid and moxifloxacin have CLSI breakpoints but no established correlation with outcome, and their results are reported here as *in vitro* information rather than as a basis for regimen construction.

Second, categorical results for agents without a validated breakpoint are only as stable as the criteria behind them. Two lines of evidence in this cohort make that concrete: the tested concentrations did not change, yet the same MIC value was assigned to different categories at different times in 18 of 41 species–agent pairs; and between-period differences were concentrated among agents whose thresholds had been revised, 6 of 11 comparisons differing for agents without a CLSI breakpoint against 2 of 21 for agents with one (Section 3.2). Our own revisions were deliberate and documented, made to align with successive CLSI standards, and the lag between publication and implementation is typical of clinical laboratories. That is precisely why the problem is general: a surveillance synthesis that pools categorical proportions across laboratories and across years assumes a stable categorization rule, and that assumption can fail even within one laboratory using one assay. A reported change in NTM resistance is uninterpretable unless the interpretive criteria applied accompany it.

Three measures would make such data comparable. MIC values rather than categories should be the unit of NTM surveillance, since they are invariant to interpretive change. Agents without a validated clinical breakpoint should be reported numerically, or withheld from the report, because issuing them as “resistant” predictably leads to drug withdrawal. And epidemiological cut-off values derived from pooled multicenter MIC distributions (Fröberg et al., 2023) offer a defensible interim framework for separating wild-type from non-wild-type populations while clinical breakpoints remain unavailable. In practical terms, primary-level laboratories should concentrate on identification to complex level and on clarithromycin and amikacin MICs, with extended incubation to day 14 for rapidly growing species, while provincial reference laboratories record numeric MICs for the full panel together with the version of the interpretive criteria applied to every run. Ethambutol requires explicit handling within this framework: primary-level laboratories should report an ethambutol MIC value rather than a category, and a high ethambutol MIC should not by itself prompt withdrawal of the drug, which occurred in 9 of the 85 MAC-PD patients here and risks creating effective macrolide monotherapy (Section 4.2). We propose a pre-specified alert rule: a year-on-year change of 10 percentage points or more in the proportion of isolates in the highest category for any agent should prompt verification of assay lot, criteria version, quality-control performance and inoculum standardization before any epidemiological interpretation.

Third, cavitary disease was associated with substantially lower odds of cure and, if that association is confirmed, would justify closer monitoring and early consideration of intensification or surgical referral, recognizing that the inverse drug-count–cure association reflects severity rather than a causal effect of regimen breadth (Kyriacou and Lewis, 2016). The 2020 guideline identifies the yield of susceptibility testing as an explicit evidence gap (Daley et al., 2020); real-world MIC-distribution data such as these help characterize the regional landscape, but prospective controlled trials remain essential before testing can be used to individualize regimens.

## 5 Conclusion

In 606 NTM isolates and 127 patients who completed at least 12 months of susceptibility-informed therapy in Shandong Province, MAC accounted for nearly three-quarters of isolates. When categories were reclassified from individual MICs against a single CLSI criterion, the four agents with validated clinical breakpoints showed near-complete *in vitro* activity against MAC: 98.9% of isolates were susceptible to clarithromycin, 99.8% to amikacin, 99.3% to linezolid and 96.2% to moxifloxacin. Categorical susceptibility testing is therefore unlikely to alter the choice of macrolide or aminoglycoside in this population, and clarithromycin is the appropriate class representative for macrolide testing, the apparent discordance with azithromycin being a property of the thresholds applied rather than of independent resistance.

For the remaining agents no validated clinical breakpoint exists, and the categories reported to clinicians rest on criteria defined by the laboratory, which were revised twice during the study period as successive CLSI standards were adopted. Categorical results for these agents are consequently not comparable between periods, whereas the MIC distributions are unaffected. NTM surveillance should be built on MIC values and should record the interpretive criteria applied with every result.

Cavitary disease was associated with substantially lower odds of cure in MAC-PD, whereas the inverse association between drug count and cure most plausibly reflects confounding by indication. *M. kansasii*-PD achieved 95.0% cure, and MABC-PD 54.5% in the absence of a strict intravenous induction phase, with recurrence after conversion concentrated in that species. Estimates for the MABC-PD and *M. kansasii*-PD subgroups rest on 22 and 20 patients respectively and should be read as descriptive. Outcome estimates derive from a completer cohort and describe that cohort only; prospective controlled studies are required to establish whether susceptibility testing can safely guide regimen individualization.

## Data Availability

The datasets presented in this article are not readily available because they contain potentially identifying clinical information. Aggregated MIC distributions and a de-identified per-patient treatment listing are provided in the Supplementary material. Requests to access the datasets should be directed to HF, fhj19850116@163.com.

## Supplementary material

Supplementary Figure 1 Age distribution of the 263 female NTM-PD patients with MAC, MABC or M. kansasii disease, in 5-year bins.

Supplementary Table 1 Laboratory breakpoint periods and the CLSI standard each was aligned to; the concentrations tested on the assay panel; and the MIC values recorded for each species–agent pair with the categories to which they were assigned.

Supplementary Table 2 Baseline characteristics of 12-month completers versus excluded patients, with standardized mean differences.

Supplementary Table 3 Complete MIC distributions by species and agent: isolates at each tested dilution, MIC range, MIC_50_, MIC_90_ and modal MIC.

Supplementary Table 4 Ethambutol MIC distribution in MAC by year of report.

Supplementary Table 5 Categories as reported at the time of testing versus CLSI categorization applied to individual MICs, for every agent with a CLSI clinical breakpoint.

Supplementary Table 6 Categorical results by laboratory breakpoint period, stratified by whether a CLSI clinical breakpoint exists.

Supplementary Table 7 Primary, parsimonious and Firth penalized-likelihood models for cure in MAC-PD.

Supplementary Table 8 Complete listing of low-MIC co-occurrence combinations of three to six agents for MAC and MABC.

Supplementary Table 9 Treatment exposure and outcome in the 127 completers, including regimen definitions and doses, adverse events, agents implicated, and a per-patient de-identified listing.
